# Timing of brain entrainment to the speech envelope during speaking, listening and self-listening

**DOI:** 10.1016/j.cognition.2022.105051

**Published:** 2022-07

**Authors:** Alejandro Pérez, Matthew H. Davis, Robin A.A. Ince, Hanna Zhang, Zhanao Fu, Melanie Lamarca, Matthew A. Lambon Ralph, Philip J. Monahan

**Affiliations:** a MRC Cognition and Brain Sciences Unit, University of Cambridge, UK; b Department of Language Studies, University of Toronto Scarborough, Canada; c Department of Psychology, University of Toronto Scarborough, Canada; d School of Psychology and Neuroscience, University of Glasgow, UK; e Department of Linguistics, University of Toronto, Canada

**Keywords:** Entrainment, Speech, Production, Perception, Self-listening

## Abstract

⁠This study investigates the dynamics of speech envelope tracking during speech production, listening and self-listening. We use a paradigm in which participants listen to natural speech (Listening), produce natural speech (Speech Production), and listen to the playback of their own speech (Self-Listening), all while their neural activity is recorded with EEG. After time-locking EEG data collection and auditory recording and playback, we used a Gaussian copula mutual information measure to estimate the relationship between information content in the EEG and auditory signals. In the 2–10 Hz frequency range, we identified different latencies for maximal speech envelope tracking during speech production and speech perception. Maximal speech tracking takes place approximately 110 ms after auditory presentation during perception and 25 ms before vocalisation during speech production. These results describe a specific timeline for speech tracking in speakers and listeners in line with the idea of a speech chain and hence, delays in communication.

## 1 Introduction

During conversation, speakers and listeners exchange information; sending signals from one brain to another via the medium of speech. Consistent with the idea of the speech chain (Denes & Pinson, 1993), the speaker's brain activity should lead the listener's due to transmission delays from the speaker to the listener, as well as other physical limitations that mediate speech communication. Among these, transmission delays of ~5 ms from the speaker to the listener are expected given the speed of sound for conversations over 1–2 m of distance. These physical delays, however, are negligible compared to the likely neural delays due to speech planning on the part of the speaker, and the neural mechanisms involved in comprehension on the part of the listener. In this paper we measure the duration of two salient neural delays that are ubiquitous during conversation: the time elapsed from speech planning to articulation during speaking, and from speech input to speech perception during listening.

Given the intrinsic nature of these delays, it might seem inevitable that there is a measurable delay in communicating an idea from a speaker to a listener during conversation. Perhaps surprisingly, however, neural observations in speakers and listeners often show instantaneous inter-brain synchronisation without measurable phase-lag (Ahn et al., 2017; Dai et al., 2018; Kawasaki, Yamada, Ushiku, Miyauchi, & Yamaguchi, 2013; Pérez, Carreiras, & Duñabeitia, 2017). In other words, consistent brain activity between the speaker and listener is evident without assuming that the listener's brain signals are delayed relative to the speaker's brain signals (e.g., Dai et al., 2018). Observations of instantaneous inter-brain synchronisation challenge the idea of a speech chain. Instead, they emphasise the predictive nature of speech communication, in particular and interactive language use, in general (Pickering & Garrod, 2021).

However, before we can conclude that verbal inter-brain synchronisation during speaking and listening, measured with high temporal resolution techniques, reflects mutual predictive processing, we need to rule out some methodological concerns. The most important concern relates to the phenomenon of brain entrainment to speech (see for review, Poeppel & Assaneo, 2020). Here, brain activity ‘tracks’ slow modulations in the overall amplitude of the auditory signal or speech envelope. The logic behind brain entrainment to speech contributing to measured inter-brain synchronisation is straightforward: Because there will be coupling between the listener's cortex and the speech, and because the speaker is also hearing their own speech (both through air and bone conduction), inter-brain coupling might simply be a by-product of concomitant brain entrainment to speech sounds in the speaker and listener. That is, instantaneous inter-brain coupling could occur because both brains are simultaneously tracking the envelope of heard speech at the same delay. However, if we observe different delays in speech tracking for the listener and the speaker, this is in opposition to the idea that inter-brain synchronisation is due to concomitant brain entrainment to speech sounds. Instead, it may support predictive accounts of inter-brain synchronisation.

It has previously been shown that inter-brain synchronisation effects remain after removing the contributions of brain entrainment to the speech envelope by using statistical procedures (e.g., Pérez et al., 2017). Here, however, we use EEG and a design that separated speaking and listening to directly assess whether delays in speech envelope tracking differ as a function of an individual's conversational role (speaker versus listener). For the listener, existing data show consistent coupling between brain activity and the speech envelope occurs at positive delays—taking the speech signal as the reference (Brodbeck, Hong, & Simon, 2018; Broderick, Anderson, & Lalor, 2019). Similar misalignment between brain activity and the speech envelope in a positive direction is also seen for amplitude modulated noise (Crosse, Di Liberto, Bednar, & Lalor, 2016) and unintelligible speech (Ding, Chatterjee, & Simon, 2014). For the speaker, neural tracking of self-produced speech could be inhibited/reduced since predicted sensory consequences of self-initiated actions are suppressed due to motor control processes, such as efferent copies (Houde & Chang, 2015; Zheng, Munhall, & Johnsrude, 2010). We might also expect that the neural processes most critical for speech production occur prior to vocalisation onset. These processes, for example, could contribute to lexical access, to phonological and phonetic encoding and to the implementation of the motor plan (corollary discharge) leading to speech production (Hickok, 2012). Thus, during speaking, we might expect maximal speech envelope tracking to occur prior to verbalisation; however, these negative delays between brain activity and speech envelope have not, thus far, been established. In summary, in the present study we investigate the lagged relationships between electroencephalographic (EEG) brain activity and the acoustic envelope during speaking and listening.

We use a paradigm in which participants listen to natural speech (Listening), produce natural speech (Speech Production), and listen to the playback of their own speech (Self-Listening), while their neural activity is recorded with EEG. We include the Self-Listening condition to (i) compare speaking and listening without stimulus differences, and (ii) setup a listening situation in which accurate predictions for upcoming speech signals are possible (as during speaking). Comparison of the Listening and Self-listening conditions can thereby assess latency shifts and other changes associated with increased predictability. Mutual information measures based on the Gaussian copula (Ince et al., 2017) are used to quantify the amount of information obtained about brain activity through observing the speech envelope (Gross et al., 2021). We analysed the peak of lagged Gaussian Copula Mutual Information (GCMI) to distinguish the timing of maximal brain entrainment to speech. The 2–10 Hz frequency range is investigated, since this covers a range of neural activity that oscillation-based speech perception models emphasise as important for speech intelligibility (Ahissar et al., 2001; Ghitza, 2011; Luo & Poeppel, 2007). Our results help delineate the time course of brain entrainment to the speech envelope during speaking and listening and carry implications for speech chain and predictive accounts of speech processing.

## 2 Methods

### 2.1 Participants

All participants were recruited from the University of Toronto community. Two English speakers (1 female) performed a simplified version of the experiment, including the EEG recording. They only participated in the Speech Production portion of the task. Their productions were used to create the initial auditory stimuli. These two participants are referred to as ‘reference subjects’ (henceforth, RS). An additional 13 English speakers (10 female; mean age: 21 years; SD = 1.6; range: 19–26) took part in all three portions of the task. All participants self-reported normal or corrected vision, normal hearing and showed typical speech/reading abilities. This was evaluated by the experimenter during the debriefing session. Participants received monetary compensation. Individual written informed consent was obtained, including permission for making their EEG publicly available. The experiment was approved by and carried out following the guidelines and regulations of the University of Toronto's Research Ethics Board.

Data exclusion criteria were defined a priori. These included technical issues during EEG acquisition (e.g., amplifier saturation, high impedances), non-fluid speech production (less than 100 words spoken during the Production task), and more than 25% of the EEG signal during a particular epoch being irrecoverable due to significant artefacts. No data were excluded due to these criteria.

### 2.2 Experimental design

**Fig. 1:**
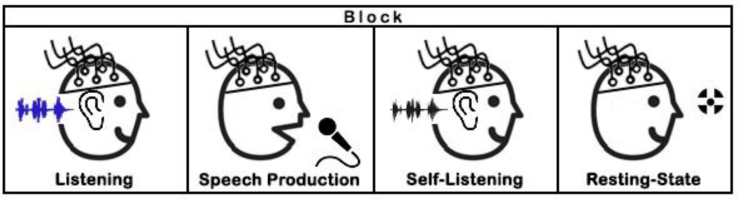
Experimental design. Conditions present in each block. In the Listening condition, participants (*N* = 13) listen to speech recordings produced by two additional participants (auditory signal represented in blue). In Speech Production, participants (*N* = 15) overtly speak about the same topic that they heard spoken about by the other speaker. During Self-Listening, participants listen to their own recorded speech (N = 15). In Resting-State, participants watch a fixation cross. Four blocks corresponding to four different topics are presented. Presentation order of the Resting-State condition is random inside the block. (For interpretation of the references to colour in this figure legend, the reader is referred to the web version of this article.)

Fig. 1 depicts the tasks used in the experimental design. The nature of each of these tasks, as well as the Evaluation part, is described below:

Listening: Attentively listening through headphones to the RS' preferences and opinions about a specific topic. The Listening task occurs once for each RS, resulting in twice the number of trials compared to other tasks. The RS presentation order was randomised.

Speech production: Speaking overtly about preferences and opinions on the same topic. The produced speech was recorded.

Self-listening: Attentively listening to the playback of the previously recorded audio file (i.e., their own speech).

Resting-state: Remaining quiet while watching a fixation cross (Thaler, Schütz, Goodale, & Gegenfurtner, 2013). The presentation order of this Resting-State task was random relative to the previous three tasks.

Evaluation: Completing a questionnaire about the content of the speech presented in the Listening task. The Evaluation took place at the end of each block. This consisted of ten multiple-choice questions with three choices each. Five questions were dedicated to each RS, and they were visually presented one-by-one. The order of the questions followed the order in which they appeared in the RS's speech. The participant responded by pressing the key corresponding to the number of the correct answer.

The beginning of each task was self-triggered. All tasks lasted 125 s, except for Evaluation, which had no time constraint. During the Speaking task, participants were provided with five questions about the topic to help structure the discourse. This template remained on the screen during the Listening task (Pérez et al., 2017; Pérez, Dumas, Karadag, & Duñabeitia, 2019). The overall experiment session was divided into four distinct blocks containing the tasks. Each block revolved around a unique topic (i.e., animals, music, movies, food). Participants were asked to speak freely, continuously, and clearly (‘like in a podcast’), to avoid reading the questions as much as possible, and to minimise body movement and the use of interjections. They were instructed to pay careful attention to the content of the speech, including their own, because this would be evaluated at the end of the trial. The instructions were presented on the screen before each task. In total, the Listening task was presented eight times (i.e., 2 PS × 4 topics), and Speech Production, Self-Listening, Resting-State, and Evaluation occurred four times.

### 2.3 Experiment setup

The experiment was conducted in a dimly lit, sound-attenuated cabin. Participants were comfortably seated facing an Alienware monitor (120 Hz refresh rate) at a distance of approximately 90 cm. The stimulation computer included a high-fidelity sound card ESSENCE STX II. A custom-written program using PsychoPy3 (Peirce et al., 2019) controlled the experiment, including the presentation of all stimuli and instructions, the recording and saving of the produced speech audio files, and finally, the sending of the digital markers to the EEG acquisition software. A BBTK USB Response Pad (Button box) was used to navigate the instructions, trigger trials, and collect responses to the questionnaire.

#### 2.3.1 EEG settings

Electrophysiological data were acquired with 32-channels actiCAP active electrodes and one-module actiCHamp amplifier (Brain Products GmbH). Electrodes were mounted on an elastic cap and placed according to the International 10–20 system. This included: Fp1/Fp2, F3/F4, F7/F8, FT9/FT10, FC1/FC2, FC5/FC6, C3/C4, T7/T8, CP1/CP2, CP5/CP6, TP9/TP10, P3/P4, P7/P8, O1/O2, Fz, Cz, Pz and Oz. The electrical Ground electrode was placed at Fpz. Signals were recorded reference-free at a sampling rate of 1000 Hz. Inter-electrode impedances were set below 20 kΩ at the beginning of the experiment. Horizontal and vertical eye movements were monitored using four additional electrooculography (EOG) electrodes connected to two BIP2AUX adapters and then to two different AUX (auxiliary) inputs of the actiCHamp. A StimTrak device and a photosensor attached to the screen monitor (data not used here) were also connected to AUX inputs. The StimTrak received the auditory signal via a pass-through connector. Thus, signals recorded in the actiCHamp amplifier included: the EEG signal and any auditory signal delivered by the headphones. To note, they were all jointly recorded on the same workspace using the PyCorder software (Brain Products, GmbH). The data were continuously acquired during the entire experiment.

The experimental settings contain essential features to ensure consistent auditory input in speech production and self-listening conditions (i.e., reducing the influence of bone-conduction) and to allow the measurement and correction of timing between the physical delivery of the audio signal and the ongoing EEG signal (i.e., no time-shift). These are, (i) the audio signal captured by the microphone is played back by the headphones with near-zero latency, (ii) all audio volumes are similar and (iii) both the auditory signal delivered to the headphones and the ongoing EEG activity are synchronously recorded (Pérez, Monahan, & Lambon Ralph, 2021).

### 2.4 EEG and audio pre-processing

The data were analysed using EEGLAB v2019.1 (Delorme & Makeig, 2004) and custom programs, all running in MATLAB R2019b (The Mathworks, Inc). First, the recorded signal was imported to EEGLAB, and channels locations were added. Then, the reference electrode standardisation technique was applied (Dong et al., 2017)**.** Subsequently, data were re-sampled (250 Hz), high-pass filtered (2 Hz), and line noise was removed. Bad channels were automatically detected (Bigdely-Shamlo, Mullen, Kothe, Su, & Robbins, 2015), removed and interpolated using spherical splines interpolation (Perrin, Pernier, Bertrand, & Echallier, 1989). Next, an artefact subspace reconstruction (ASR) algorithm (Bigdely-Shamlo, Mullen, Kreutz-Delgado, & Makeig, 2013; Mullen et al., 2013; Mullen et al., 2015) was used to remove high amplitude artefacts, using a variance threshold of 10 (Chang, Hsu, Pion-Tonachini, & Jung, 2018). Portions of the data that were impossible to reconstruct due to the presence of multiple artefacts were marked. Then, data were epoched, and an adaptive mixture independent component analysis (AMICA) technique was applied (Delorme, Palmer, Onton, Oostenveld, & Makeig, 2012; Palmer, Kreutz-Delgado, Rao, & Makeig, 2007). For the independent components, the single equivalent current dipoles were estimated and anatomically localised (Oostenveld & Oostendorp, 2002), including searching for and estimating symmetrically constrained bilateral dipoles (Piazza et al., 2016). A number of high probability brain components (mean = 9, SD = 3.2, range: 4–14) were automatically selected (Pion-Tonachini, Hsu, Chang, Jung, & Makeig, 2018). Finally, the timing of the EEG markers was adjusted to correctly synchronise with the auditory stimulus (mean time-shift: 9 ms, SD = 9). Synchronisation was performed by computing the cross-correlation function between the auditory signal recorded with the EEG via StimTrak and the audio file downsampled to match the sampling frequency of the EEG. This allowed for millisecond-level precision between the EEG signal and the auditory stimulus that was delivered. In summary, the pre-processing separated channel-level data from each participant into a sum of maximally independent component subspaces with neural origins.

Speech envelopes were extracted from the high fidelity audio recordings by using half-wave rectification and smoothing with a second-order low-pass filter with a cut-off frequency of 30 Hz (Biesmans et al., 2015). The envelopes were downsampled to 250 Hz and band-pass filtered in those frequencies to be analysed using a third-order Butterworth filter. The same filtering method was applied to the EEG signals already aligned with the envelope.

### 2.5 Analysis

The Gaussian Copula Mutual Information (GCMI) measures related information content between two variables that could be multidimensional (Ince et al., 2017). The method is qualitatively different from measures of phase consistency between oscillators that assume phase synchronisation as the indication of a link between biological systems (Strogatz, 2003). It uses a rank-based transform, is robust to outliers, and makes no assumptions of the marginal distributions of each variable; therefore, it can be applied to any continuous-valued data. It provides results with a meaningful common effect size (bits). Specifically, we used the GCMI to describe the time evolving covariations between the speech signal and the brain activities of speaker and listener. The GCMI employed here used an instantaneous temporal derivative (gradient) together with the raw signal values, and it was calculated over a range of delays (henceforth, lagged GCMI) in a procedure similar to cross-correlation.

The lagged speech envelope tracking was estimated by calculating the lagged GCMI between individual brain signals and the speech envelope for each subject and speech condition. The time window for estimation was from −400 ms to 400 ms in 4 ms intervals. Negative latencies indicate that the auditory signal precedes the EEG signal, while positive lags indicate that the brain activity follows the auditory signal. Zero lag is when the auditory and EEG signals are synchronous.

#### 2.5.1 Scalp-level

Scalp-level lagged speech envelope tracking was calculated between the individual scalp-level brain signals (EEG channel array) and the speech envelope. To test for a significance requirement of 95%, 1000 surrogates were created for each participant and condition. The number of surrogates (M = 1000) was chosen from M = *K*/α – 1 where *K* = 50 and α = 0.05 (see Schreiber & Schmitz, 2000). Surrogates were obtained by perturbing the speech envelopes, while preserving autocorrelation by using the cyclic phase permutation method (Lancaster, Iatsenko, Pidde, Ticcinelli, & Stefanovska, 2018). The perturbed speech envelopes were paired with intact scalp-level EEG array and the lagged GCMI calculated. Then, for each latency, we compared if the group mean GCMI value averaged across channels was larger than the maximum null mean GCMI obtained for any latency and channel.

#### 2.5.2 IC-level

IC-level lagged speech envelope tracking was calculated between the speech envelope and the individual high probability brain component signals (IC components obtained from AMICA). For each participant and condition, we identified the exact latency and IC containing the largest GCMI value over the time–lag range. In other words, we searched for the latency showing the peak amplitude in the speech envelope tracking. Then, those latency values were compared across conditions.

## 3 Results

### 3.1 Behavioural

All participants consistently performed above chance (i.e., > 33.3%) in responding to the content of the presented speech stimuli. The mean percentage of correct responses obtained in the Evaluation task was 88.3% (SD = 10.6). Mean accuracy did not significantly differ across reference speakers (RS) or Topics. These results indicate that all participants were paying attention.

### 3.2 Lagged speech envelope tracking

**Fig. 2:**
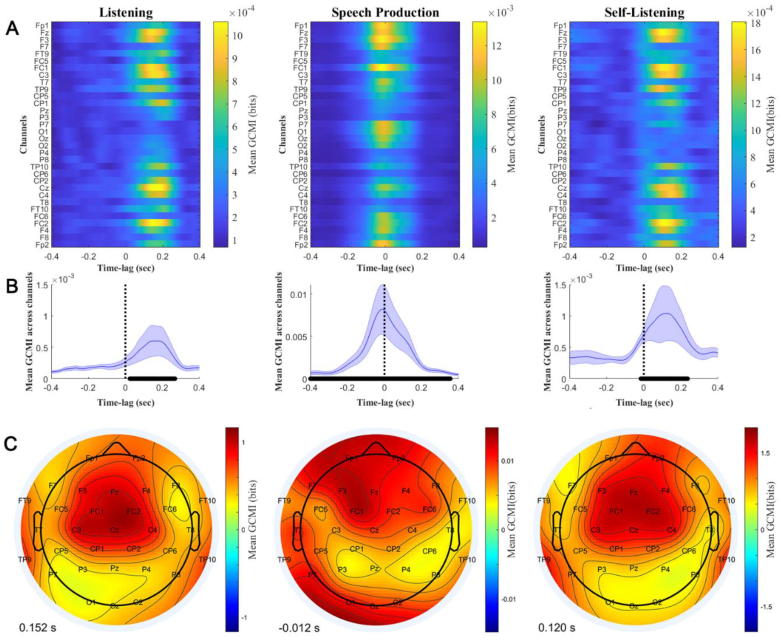
Mean lagged speech envelope tracking estimated at the scalp-level and grouped by condition. Negative times indicate the audio signal preceding the EEG signal and positive lags indicate that the brain activity follows the auditory signal. Zero lag is when the auditory and EEG signals are synchronous. Each column represents a condition. Panel A contains the channel-time-lag representation of the sample average. Colour scale represents the magnitude of the GCMI value (bits). Panel B contains the mean (blue line) and standard deviation (blue shadowed area) when collapsing across channels. The black dots on the x-axis indicate the lags in which the GCMI values are increased (95% of confidence). Panel C contains the response topography at the latency showing the maximum GCMI value, which is indicated at the bottom. (For interpretation of the references to colour in this figure legend, the reader is referred to the web version of this article.)

First, we explored the lagged speech envelope tracking at the scalp-level. Fig. 2 shows the group average of the lagged GCMI values grouped by condition. Panel A contains all channels, and Panel B represents the mean and the standard deviation after averaging across channels. Black dots on the X-axis indicate latencies showing increased GCMI values at a 95% significance threshold. Fig. 2B suggests that speech envelope tracking peaks differently for listening and speaking, with positive delays for speech perception (152 ms for Listening and 120 ms Self-Listening) and close to zero delays for speech production (−12 ms). The animation provided in Supplementary Material 1 shows the time evolving scalp distribution of GCMI values and their magnitudes for each experimental condition. The Speech Production condition shows a fronto-posterior scalp topography at the peak amplitude of the otherwise largest GCMI values. In contrast, for the listening conditions, the maximal MI is evident at central areas.

We compared the magnitude of the lagged GCMI values averaged across channels during Listening and Self-Listening using a Wilcoxon signed rank test. The test indicates that the GCMI values for the Self-Listening are larger for the interval containing the maximum values on both condition (from 0 to 172 ms, all ps > 0.05, false discovery rate corrected). We also compared the variances using a Levene's Test. The variances are significantly different, larger for Self-Listening, for those delays containing the peak values (from 36 to 152 ms, all ps < 0.05, false discovery rate corrected).

**Fig. 3:**
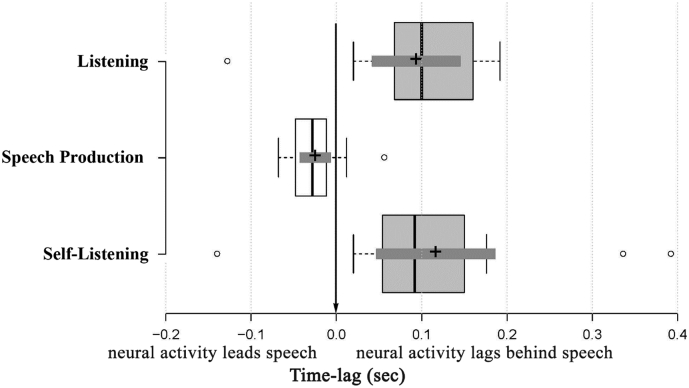
Latencies corresponding to the maximum speech envelope tracking estimated by condition. Positive time values represent the time elapsed after the auditory stimulus was recorded, and negative values represent times before the start of the auditory recording. Center lines show the medians over participants; box limits indicate the 25th and 75th percentiles as determined by BoxPlotR (Spitzer, Wildenhain, Rappsilber, & Tyers, 2014); whiskers extend 1.5 times the interquartile range from the 25th and 75th percentiles, outliers are represented by dots; crosses represent sample means; bars indicate 95% confidence intervals of the means. Participant counts: *n* = 15, 15 and 13 sample points for Self-Listening, Speech Production and Listening, respectively.

Next, we explored the time latencies at which maximal speech envelope tracking is shown in Independent Component (IC) data. Fig. 3 shows the statistics for those latency values showing the strongest speech envelope tracking, grouped by condition. Differences in peak latencies between these results resemble those obtained with scalp-level data and shown in Fig. 2B. For conditions in which participants were producing speech, maximal GCMI values occurred on average 25 ms (SD = 32) *before* the onset of the auditory stimuli (i.e., neural activity anticipated the speech envelope). When participants were listening to speech, the maximal GCMI values occurred *after* delivering the auditory stimulus to the participants. The average time lag between speech and neural activity is 117 ms (SD = 126) when participants were listening to others and 94 ms (SD = 86) for self-listening. Despite the numerical difference in latency, there was no statistically significant differences between the time-lag of maximum GCMI values when comparing the Listening and Self-Listening conditions (Wilcoxon signed rank test, *p* = .26; BF_01_ = 3.74, i.e., moderate statistical support for the null hypothesis of no difference in latency between Listening and Self-Listening).

However, the latencies of the peak GCMI values for the two listening conditions were both significantly delayed compared to the Speech Production condition. This is confirmed by a one-tailed Wilcoxon signed rank test (for Listening: Z = −2.8, *p* = .0026, power = 90% and for Self-Listening: Z = −3.01, *p* = .0013, power = 66%). This test further suggests that the latency of maximal GCMI values for Speech Production are significantly smaller than zero (Z = −2.39, *p* = .0085, power = 58%), meaning that they preceded the onset of vocalisation.

**Fig. 4:**
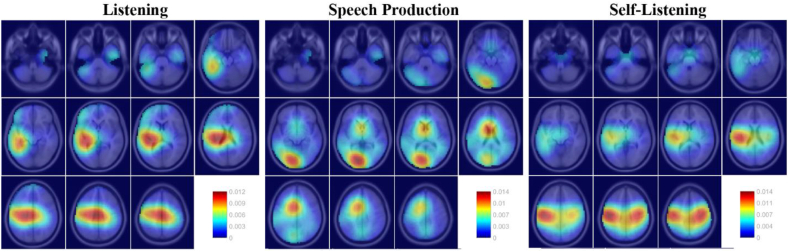
Dipole density plot of those ICs showing the maximum speech envelope tracking, grouped by condition. The colour scale represents the dipole density values, which are normalised such that the voxels of the brain sum to one.

Fig. 4 presents the tiled dipole density plot (Bigdely-Shamlo et al., 2013) (Montreal Neurological Institute's canonical template) associated with the ICs containing the maximum GCMI values, grouped by condition. This visualization allows us to better understand the neural localisation of the ICs containing maximal speech envelope tracking in each condition. For the two listening conditions, the projections of single equivalent dipoles are localised at left-lateralised auditory cortex and premotor cortex. In contrast, Speech Production shows a qualitatively different pattern of dipole localisations, clustering in left-occipital/cerebellar and middle central brain regions. These qualitative differences suggest different anatomical localisation for tracking the speech envelope between the Listening and the Production conditions.

## 4 Discussion

Using EEG, we investigated the latency at which maximal neural tracking of speech envelopes is observed during speech production, listening, and self-listening. We showed a specific timeline for speech tracking in speakers and listeners such that brain activity is linked to slow amplitude modulation frequencies conveyed by the speech envelope that are necessary for speech intelligibility. Results indicate that maximal neural tracking during listening takes place, on average, about 110 ms after speech presentation. Moreover, equivalent latencies were obtained when listening to speech that was previously spoken by the same participant (Self-Listening) or by a different participant (Listening), two situations that have been shown to induce equivalent temporal lobe activation in functional imaging studies (McGuire, Silbersweig, & Frith, 1996; Price et al., 1996). For the Self-Listening condition, multiple levels of language representation (e.g., syntax, lexical items, prosody, phonetics) are putatively predictable since there is a short time elapsed between participants' production and the replay. The electrophysiological results here nonetheless suggest equivalent neural timing for processing other's speech and self-produced speech, despite these differences in predictability (but see Maslowski, Meyer, & Bosker, 2018). This observation argues against the suggestion that increased prediction accelerates neural speech-tracking; however, prediction might affect the strength of entrainment. Enhancements in the strength of speech tracking with no change in latency have been reported for brain entrainment to speech during increased top-down focal attention (Lesenfants & Francart, 2020). Interestingly, larger magnitudes of speech tracking (larger GCMI values) were observed during Self-Listening and there is more between-subject variance during Self-Listening as compared to Listening. This is in line with recent studies showing individual differences in the interplay between perceived and produced speech (e.g., Assaneo et al., 2019). That said, we are cautious in drawing conclusions from this observation of the variance as the number of trials are not equivalent between conditions: The Listening task has twice the number of trials as the Self-Listening task.

The localisation of the neural sources associated with listening closely matched brain regions previously shown to be involved in speech tracking, including inferior frontal and auditory regions in which cerebro-acoustic coherence is modulated by intelligibility (Peelle, Gross, & Davis, 2013), as well as the precentral gyrus for which top-down connectivity to auditory areas has previously been shown during speech comprehension (Park, Ince, Schyns, Thut, & Gross, 2015). Our results show a positive delay in coupling between these regions and the speech envelope which is in line with other reports, and evidence showing that the phonetic information is processed approximately 114 ms after the speech signal (Brodbeck et al., 2018; Broderick et al., 2019; Crosse et al., 2016; Ding et al., 2014; Gross et al., 2013).

Conversely, results from the Speech Production task indicate that maximal ‘speech envelope prediction’ takes place approximately 25 ms before vocalisation. This is in line with evidence showing that auditory modulation occurs before self-generated sounds (Stenner, Bauer, Heinze, Haggard, & Dolan, 2015). The neural sources associated with the EEG components showing maximal tracking include the supplementary motor area, a key region for speech production (Hertrich, Dietrich, & Ackermann, 2016) and posterior and inferior brain regions, including the occipital cortex and/or cerebellum. Due to the smooth spatial resolution of EEG source localisation, it is not possible to reliably distinguish occipital and cerebellar dipole localisations (Andersen, Jerbi, & Dalal, 2020). This latter localisation seems more likely a priori given the proposed role for the cerebellum in predicting the auditory sensory consequences of speaking (Guenther, Ghosh, & Tourville, 2006; Hickok, 2012).

From a mechanistic perspective, there are various cognitive processes that must be implemented by neural systems for speech production before sound emission (e.g., semantic, lexical and motoric processing of speech) (Hickok, 2014). After speech production, though, neural responses to the self-generated sensory consequences of speaking are attenuated for approximately 200 ms (Toyomura, Miyashiro, Kuriki, & Sowman, 2020; Wang et al., 2014). Taken together, these results of maximal speech tracking after auditory presentation during perception and before vocalisation during speech production support recent fMRI findings indicating alignment of the speaker's articulatory system and the listener's auditory system (Liu et al., 2020); however, by providing EEG estimates of the temporal dynamics of brain signals linked to the physical acoustic characteristics of the speech signal, we confirm that the timing of neural responses is in line with accounts of communication based on the idea of a speech chain linking the brains of speakers and listeners (Denes & Pinson, 1993).

The findings here have further implications for previous observations of synchronised neural activity during speaking and listening (Ahn et al., 2017; Dai et al., 2018; Kawasaki et al., 2013; Pérez et al., 2017). The differential delays seen in speaking and listening suggest that instantaneous inter-brain synchronisation is not exclusively mediated by the physical properties of the shared (auditory) stimulus. In other words, given the neural delays observed in speaking and listening, we might expect inter-brain synchronisation during a conversation to be delayed by at least the sum of these two delays (i.e. 25 + 94 = 119 ms, based on delays during speaking and self-listening). Some other mechanism, such as mutual active inference processes, may therefore be necessary to explain instantaneous inter-brain synchronisation during conversational interactions. In a predictive processing framework, successful mutual predictions that facilitate communication between interlocutors are achieved when their internal representations or situation models are aligned (zero lagged) (Friston and Frith, 2015a, Friston and Frith, 2015b). In the case of spoken language considered as cooperative joint action, predictions are achieved through linguistic alignment that takes place at multiple levels: from low-level acoustic features, through lexical and syntactic representations, to situation models (Garrod & Pickering, 2009; Pickering and Garrod, 2004, Pickering and Garrod, 2013). As such, if two people use the same neural mechanisms to predict what they are going to hear and to enact those predictions in the speech that they are producing, it does not matter who is speaking and who is listening. Since both are determining what they and the interlocutor are likely to say next, they are jointly predicting the same things, and their brains will be synchronised throughout (Schoot, Hagoort, & Segaert, 2016).

A possible limitation of the current study is that EEG responses in the speech production condition could be confounded by muscle activity associated with articulatory movements of the occipitalis muscle, mouth, or tongue. While we made every effort to remove non-neural signals from the EEG data using ASR algorithm and AMICA technique, these muscle artefacts might explain the increased magnitude of GCMI in speech production compared to speech perception, though, we would argue, not the differential timing. Future electrophysiological studies addressing the time course of brain entrainment to speech during production and listening would also benefit from more detailed analysis of data from anatomically (or functionally) constrained auditory and motor brain regions that can provide evidence for source-specific features of neural entrainment.

In summary, our study helps to delineate the time course of brain entrainment to the speech envelope during speaking and listening. The results are consistent with the idea of the speech chain, that is, the timing of maximal speech envelope tracking differs between the speaker and the listener. These findings raise important questions concerning the mechanisms that support synchronisation of brain activity by speakers and listeners during conversation that should be addressed in future studies.

The following are the supplementary data related to this article.

Supplementary video 1: Animation showing the time evolving scalp distribution of GCMI values and their magnitudes for each experimental condition Supplementary video 1

## Funding

AP received funding from the European Union's Horizon 2020 Research and Innovation Programme under the Marie Sklodowska-Curie grant agreement No 840885. MHD received intramural funding from the 10.13039/501100000265Medical Research Council (MC_UU_0005/5). RAAI was supported by the 10.13039/100010269Wellcome Trust [214120/Z/18/Z]. MALR was supported by an ERC Advanced grant (GAP: 670428 - BRAIN2MIND_NEUROCOMP) and intramural funding from the 10.13039/501100000265Medical Research Council (MC_UU_00005/18). This work received funding from the Social Sciences and Humanities Research Council (SSHRC) of Canada (IDG 430-15-00647), and the Natural Sciences and Engineering Council (NSERC) of Canada (RGPIN-2017-06053) granted to PJM.

## CRediT authorship contribution statement

**Alejandro Pérez:** Conceptualization, Methodology, Software, Validation, Formal analysis, Investigation, Data curation, Writing - original draft, Writing - review & editing, Visualization, Supervision, Funding acquisition. **Matthew H. Davis:** Conceptualization, Methodology, Writing - review & editing. **Robin A.A. Ince:** Methodology, Software, Writing - review & editing. **Hanna Zhang:** Software, Investigation, Project administration. **Zhanao Fu:** Investigation, Project administration. **Melanie Lamarca:** Investigation, Project administration. **Matthew A. Lambon Ralph:** Conceptualization, Writing - review & editing, Supervision. **Philip J. Monahan:** Conceptualization, Resources, Writing - review & editing, Supervision, Funding acquisition.

## Declaration of Competing Interest

None.
